# The clinical, pathological, and genetic characteristics of lipid storage myopathy in northern China

**DOI:** 10.55730/1300-0144.5431

**Published:** 2022-05-12

**Authors:** Jingzhe HAN, Shan LU, Xueqin SONG, Guang JI, Yanan XIE, Hongran WU

**Affiliations:** 1 Department of Neurology, the Second Hospital of Hebei Medical University, Shijiazhuang, Hebei, China; 2 Department of Neurology, Harrison International Peace Hospital, Hengshui, Hebei, China; 3 Institute of Cardiocerebrovascular Disease, Shijiazhuang, Hebei, China; 4 Neurological Laboratory of Hebei Province, Shijiazhuang, Hebei, China; 5 Department of Cardiology, the Second Hospital of Hebei Medical University, Shijiazhuang, Hebei, China

**Keywords:** Lipid storage myopathy, clinical manifestation, pathological staining, gene mutation, *ETFDH*

## Abstract

**Background/aim:**

The lipid storage myopathy (LSM) diagnosis is based on the patient’s clinical manifestations and muscle pathology. However, when genetic testing is lacking, there is a high rate of misdiagnosis of the disease. This study aimed to investigate the clinical and pathological features of genetically diagnosed LSM in northern China, analyze genetic mutations’ characteristics, and improve the LSM diagnostic rate.

**Materials and methods:**

Twenty patients with LSM diagnosed were collected; meanwhile, the clinical data, muscle samples, and routine pathological staining of muscle specimens were collected. The morphological changes of muscle fibers were observed under an optical microscope.

**Results:**

Among the included patients, 18 cases had *ETFDH (HGNC ID: 3483)* mutations, and two had *PNPLA2* mutations. Family pedigree verification was performed on three patients with heterozygous mutations in the *ETFDH* gene complex. Histopathological staining showed that all patients had fine vacuoles in the muscle fibers, and some of them merged to form fissures, and the lipid droplets increased in cells. After therapy, 18 patients were associated with a favorable prognosis, and two patients were ineffective with the treatment of neutral lipid storage myopathy (NLSDM) caused by *PNPLA2* mutation.

**Conclusion:**

The clinical manifestations of LSM are complex and diverse, mainly manifested by proximal muscle weakness and exercise intolerance in the extremities. The pathological images of LSM muscles are abnormal storage of lipid droplets in muscle fibers, primarily involving type I fibers. The LSM patients were mainly multiple acyl-CoA dehydrogenase deficiency (MADD) caused by the *ETFDH* gene mutation. It is necessary to perform an accurate typing diagnosis of LSM.

## 1. Introduction

Lipid storage myopathy (LSM) is a group of autosomal recessive myopathy caused by abnormal fat metabolism, characterized by abnormal fat storage of intramuscular fiber [1, 2]. The clinical manifestations of LSM are complex and diverse. Its main manifestations are volatility or progressive proximal muscle weakness, exercise intolerance, and rhabdomyolysis, involving multiple systems, such as the heart, liver, and kidney [3–5].

The leading causes of LSM include primary carnitine deficiency (PCD), multiple acyl-CoA dehydrogenase deficiency (MADD), neutral lipid storage diseases with ichthyosis (NLSDI), and neutral lipid storage diseases with myopathy (NLSDM). Multiple mutation genes, such as *SLC22A5, ETFA/B, ETFDH, ABHD5*, and *PNPLA2*, are involved [6,7]. The *ETFDH* gene encodes the electron transfer flavoprotein-ubiquinone oxidoreductase (ETF: QO); the primary function of this protein is to transfer electrons carried by ETF to coenzyme Q10 after redox, enter the mitochondrial oxidation respiratory chain, and finally be used for the production of ATP [8]. The *ETFDH* gene mutations cause short ETF: QO enzyme activity, which can cause damage the process of electron transfer to the respiratory chain. Subsequently, it further causes flavin protein-dependent dehydrogenase dysfunction and leads to insufficient energy production and fatty acid accumulation in muscle cells. Electrons leak into the mitochondrial matrix, forming many reactive oxygen species (ROS) in the cell; in turn, ROS induce abnormal oxidative stress in the cell, affecting the function and structure of mitochondria [9]. The patatin-like phospholipase domain-containing protein 2 (*PNPLA2*) gene mutations cause NLSDM [10]. Peripheral blood smears in patients with NLSDM showed fat droplets (Jordan bodies) in multinucleated granulocytes. The PNPLA2 gene encodes adipose triglyceride lipase (ATGL), the rate-limiting enzyme of the fat mobilization pathway. The primary function of ATGL is to hydrolyze triglycerides (TG) into triglycerides and free fatty acids [11]. The PNPLA2 gene mutations lead to insufficient ATGL enzyme activity, causing TG breakdown and deposition in different tissues and cells, leading to an inadequate supply of intracellular energy [12].

Studies found that the riboflavin (vitamin B2) responsive multiple acyl-CoA dehydrogenase deficiencies (RR-MADD) caused by *ETFDH* mutation and the clinical symptoms of patients treated with riboflavin can be significantly improved [13,14]. Although research on the genetic diagnosis of LSM has been carried out, understanding the disease is still insufficient [15]. Currently, most clinicians only confirm the diagnosis of LSM based on the patient’s clinical manifestations and combined with muscle pathology. However, the level of biochemical and molecular diagnosis of LSM has not improved, and the clinical misdiagnosis rate of patients is high [16]. Therefore, an accurate classification diagnosis of LSM patients is necessary to guide clinical treatment.

This study aims to investigate the clinical and pathological features of genetically diagnosed LSM patients in northern China and try to provide a theoretical basis for the diagnosis of LSM.

## 2. Materials and methods

### 2.1. Patient’s selection

Twenty patients with LSM diagnosed by muscle pathology and gene detection were collected from the Second Hospital of Hebei Medical University from January 2005 to December 2017. The patient’s clinical data were collected, including gender, age, first symptoms, limb muscle strength, system involvement, laboratory tests, and electromyography detection. In this article, the patients were given fresh morning urine, sent for organic acid analysis; and three drops of venous blood were taken for amino acid and acylcarnitine analysis of genetic metabolic diseases. This study was reviewed by the Ethics Committee of The Second Hospital of Hebei Medical University. All patients signed an informed consent form and underwent open muscle biopsy under local anesthesia.

### 2.2. Acquisition and processing of muscle specimens

After the patients and their families agreed and signed the informed consent form, the open muscle biopsy was performed under local anesthesia. The surgical site includes the biceps, deltoid, quadriceps, or gastrocnemius. The fresh muscle specimens were taken for embedding and quickly frozen in liquid nitrogen precooled isopentane. The frozen muscle specimens were placed in a cryostat at −22 °C, and rewarmed for about 30 min to make frozen sections with a thickness of 8μm. The remaining samples were stored in a −80 °C ultra-low temperature freezer.

The disease’s diagnosis includes: (1) Muscle biopsy reveals many lipid droplets in muscle fibers and excludes secondary muscle fat deposits such as mitochondrial myopathy and steroid myopathy. (2) There is no prominent lipid droplet deposition in muscle pathology. Still, genetic examination results suggest that acyl-CoA dehydrogenase, very long chain (*ACADVL, HGNC: 92*), or carnitine palmitoyltransferase II deficiency *(CPT-2, HGNC: 2330)* gene mutation.

### 2.3. Specimen acquisition and genetic testing

We recorded each patient’s family history in detail and queried the mutation information based on the database for Human Gene Mutation Database (HGMD), ESP, 1000g, and ExAC. Protein function was predicted using comprehensive bioinformatics protein function prediction software such as REVEL, SIFT, PolyPhen_2, Mutation Taster, and GERP+. The pathogenicity of de novo mutations was finally analyzed, relying on family validation and the 2015 ACMG guidelines. All the included LSM patients received 2mL of peripheral venous blood, which was used for next-generation sequencing analysis.

### 2.4. Pathological staining for muscle specimens

Frozen sections were stained with hematoxylin-eosin (HE) staining, Oil-Red-O (ORO) staining, Modified Gomori Trichrome (MGT) staining, NADH-tetrazolium reductase (NADH-TR) staining, succinate dehydrogenase (SDH) staining, nonspecific esterase (NSE) staining, adenosine triphosphatase (ATPase, pH 4.5, pH 10.2) staining, and periodic acid-Schiff (PAS) staining. The morphological changes in muscle fibers were observed under a light microscope.

### 2.5. Statistical methods

Data statistics processing was performed using SPSS 21.0 software. Measurement data were expressed as mean ± standard deviation (X ± SD). The 25% and 75% intervals of the median and interquartile intervals describe nonnormally distributed data. Count data were expressed as the number of observed indicators and the percentage of the total. The t-test was used for two-way comparisons of measures that conformed to a normal distribution; the nonparametric test was used for two-way comparisons of measures that did not conform to a normal distribution. A statistically significant difference was considered as p < 0.05.

## 3. Results

### 3.1. Genetic test

Among the 20 patients, 18 (90%) of the *ETFDH* gene mutations were carried out, and 2 (10%) of the *PNPLA2* gene mutations were held. Fourteen patients had a biallelic mutation in the *ETFDH* gene, two patients had mutations in the *ETFDH* gene, two patients had mutations in the *ETFA* and *ETFDH* genes, one patient had a homozygous mutation in the *PNPLA2 (HGNC:30802)* gene, and one patient had a mutation in the *PNPLA2* gene (Table 1).

Family pedigree verification was performed on three patients with compound heterozygous mutations in the *ETFDH* gene. The result shows that two *ETFDH* gene mutations were from both parents; one patient’s parents had died, the children of the patients carried different *ETFDH* genes, and the genetic pattern was consistent with autosomal recessive inheritance.

### 3.2. Clinical features of patients with LSM

#### 3.2.1. Demographic characteristics

**Figure 1:**
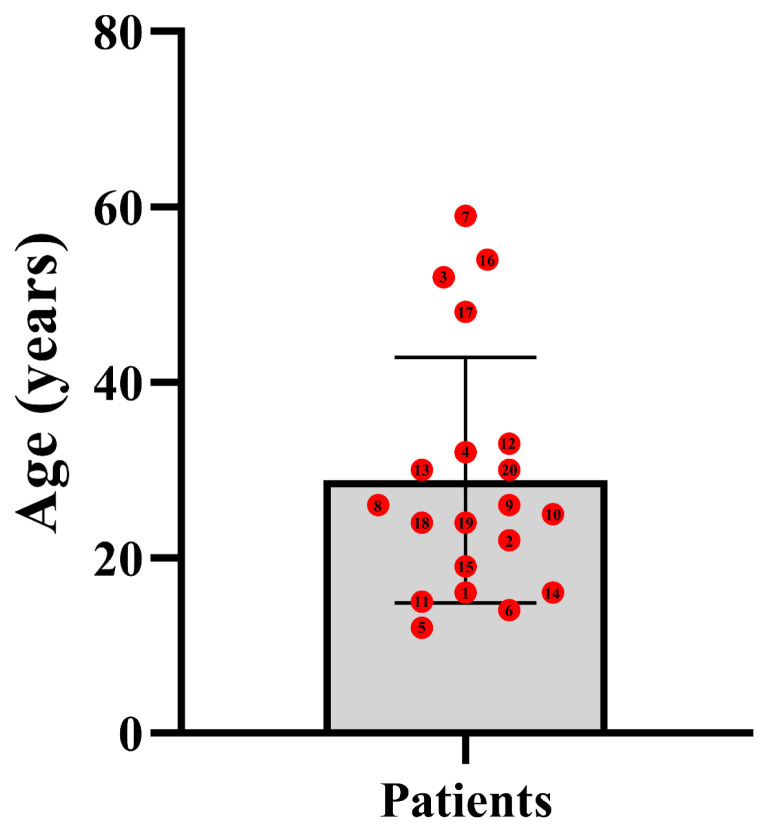
The onset age distribution of LSM patients.

Among the 20 patients, ten were male (50%). The age range of onset was 8–48 years old. There is no difference between men and women in the period of illness (p = 0.715). The patient’s age of onset is concentrated between 10–30 years old, accounting for 63.1% (12/19) (Figure 1). The patients in the article on LSM were insidious onset, with a chronic disease course ranging from 1 to 7 years (Table 2).

#### 3.2.2. Patient’s course and family history

All patients were occult seizures, with a chronic course ranging from one month to seven years. There were three (15%) family history cases among the patients; 17 (85%) were sporadic cases.

#### 3.2.3. First symptoms

In the patients, there have eight cases with lower extremity weakness (8/20, 40%), seven cases with limb weakness (7/20, 35%), three cases with upper limb weakness (3/20, 15%), and one case with poor appetite (1/20, 5%) (Table 2).

#### 3.2.4. Important symptoms with diagnostic value

In all included patients, there have 12 cases with exercise intolerance (12/20, 60%), six cases with masticatory muscle weakness (6/20, 30%), two cases with dysphagia (2/20, 10%), two cases with respiratory muscle weakness (2/20, 10%), and six cases with myalgia (6/20, 30%). The significant signs of diagnostic value included 16 (16/20, 80%) cases with decreased muscle strength and five (5/20, 25%) cases with weakness when lifting their head. In the patients with muscle weakness, there are 15 cases with symmetric (15/20, 85%), two cases with asymmetric (2/20, 10%), 11 cases with proximal weakness (11/20, 55%), one case with distal weakness (1/20, 5%), seven cases with proximal and distal weakness (7/20, 35%), and one case with normal function (1/20, 5%) (Table 2).

#### 3.2.5. Predisposing factors

Ten patients had clear induced aggravation factors: six cases were fatigue, two cases were infected, one case was caught a cold, and one case was due to fast. Other system involvement, including five cases with heart involvement (5/20, 25%), mainly manifested as sinus tachycardia. The digestive system involved five patients (5/20, 25%), primarily manifested as nausea and vomiting (Table 2).

### 3.3. Laboratory diagnosis results

#### 3.3.1. Serum CK and uric acid

The study’s serum creatine kinase (CK) value was detected in 19 patients; among the patients, 17 cases had an increase (17/19, 89.5%), with a mean of 3753.71 ± 6156.26 U/L (The normal reference range of serum CK is 18.0–198U/L). Ten uric acid cases were detected, and 8 cases (80%) of patients with uric acid were found to have an average value of 655.30 ± 351.80 U/L (normal blood uric acid: 149–416 μmol/L for adult males; 89–357 μmol/L for females) (Table 2).

#### 3.3.2. Electromyography

In this study, electromyography showed normal in 5 patients (31.25%, 5/16), myogenic injury in 10 (62.5%, 10/16) patients, and neurogenic injury in 5 (31.25%, 5/16) patients (Table 2).

#### 3.3.3. Urine analysis

A total of six patients were screened for blood and urine metabolism in this study. Three patients showed glutaric aciduria, one patient had increased multiple lipoylcarnitine (C8–C16) in blood, and two patients did not show any significant abnormalities (Table 2).

#### 3.3.4. Histopathological staining

**Figure 2:**
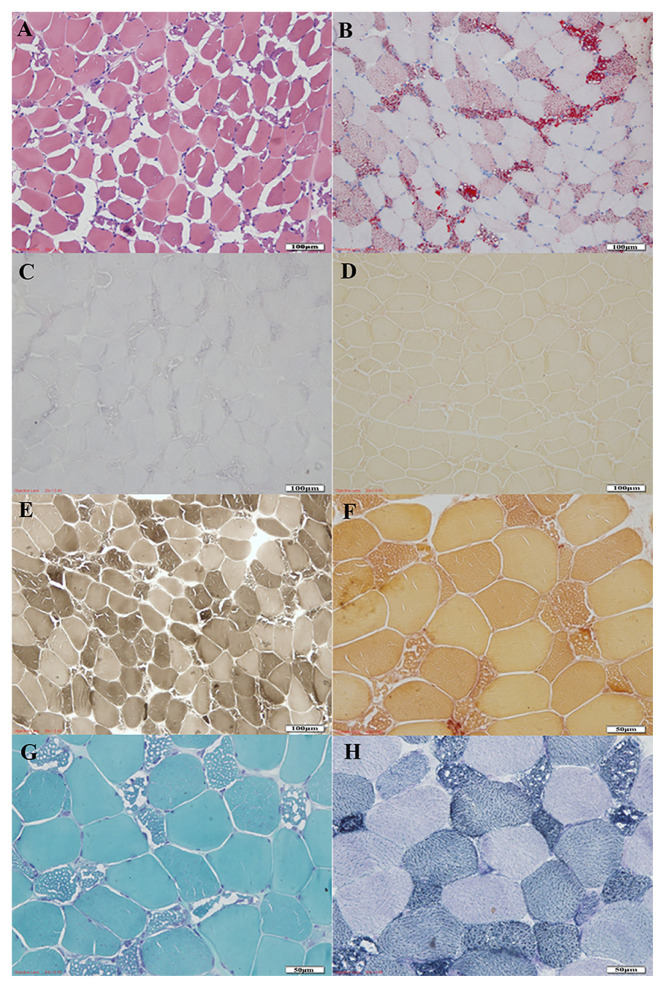
The results of characteristic histologic. (A) Hematoxylin-eosin staining in standard control (200×); (B) Oil-Red-O staining in standard control (200×); (C) Succinate dehydrogenase staining in vacuolar muscle fibers and many atrophic fibers (200×); (D) Acid phosphatase staining in lipid droplets storage of the myofibers (200×); (E) ATPase staining in the vacuolated appearance of predominantly type 1 muscle fibers (PH = 4.5, 200×); (F) Nonspecific esterase staining in deeply stained in atrophic and degenerative muscle fibers (200×); (G) Modified-Gomori-Trichrome staining in ragged red fiber (400×); (H) Nicotinamide adenine dinucleotide staining in deeply stained in atrophic and degenerative muscle fibers (400×).

All patients observed small vacuoles in the muscle fibers, and some merged to form fissures. Muscle fibers appeared as fissures or fine vacuoles in hematoxylin-eosin (HE) staining, with a marked increase in lipid droplets in ORO staining (Figures 2A, 2B). The SDH staining results of seven patients showed that the oxidase distribution of some muscle fibers was uneven, the edges were intensely stained, or the oxidase was partially atrophied. The muscle fibers were deeply stained (Figure 2C). In eight patients, the vacuolar-like muscle fibers were mainly typed I; in 12 cases, both muscle fibers showed an abnormal increase in lipid droplets (Figures 2D–2G). NADH-TR staining in nine patients showed an uneven distribution of oxidases in some muscle fibers and deep staining at the edges or partially atrophic muscle fibers (Figure 2H).

### 3.4. Treatment and prognosis

All patients were given riboflavin (30–120 mg/d) + coenzyme Q10 (150–500 mg/d) + carnitine treatment. After 7 to 14 days of treatment, the clinical symptoms began to improve in 18 (90%) patients with a more favourable prognosis. After 1 to 1.5 months, patients’ muscle strength was significantly recovered; after 1 to 3 months, most patients’ physical labour or exercise ability returned to normal, and a few patients still do not tolerate high-intensity physical activity. However, the treatment with NLSDM patients caused by *PNPLA2* mutation was ineffective (2/20). Blood uric acid levels decreased to varying degrees, and six patients (6/10) returned normal. Two patients underwent a review of muscle pathology after treatment and found that the muscle fibers’ lipid droplets decreased or even disappeared. After one year of follow-up, most patients were given riboflavin for three months and discontinued without recurrence. Two patients may experience muscle weakness after infection or fatigue, and the symptoms may be relieved after riboflavin supplementation.

## 4. Discussion

The disease of LSM can occur at any age, characterized by occult onset. In this study, 20 LSM patients were collected, the incidence rate of males and females was 1:1, and the age of onset was 8–59 years old, with an average of 26.05 ± 13.71 years old. The normal life of all patients can be affected, and most concentrated in 10–30 years old.

The clinical manifestations of LSM are complex and diverse, mainly manifested as volatility or progressive proximal muscle weakness and exercise intolerance [17, 18]. The LSM patients were insidious onset, with a chronic disease course ranging from 1 to 7 years. Generally, the first symptom in patients was mainly proximal muscle weakness. Elevated muscle enzymes and anorexia are relatively rare early symptoms in patients. The muscle weakness symptoms were symmetrical; only two patients had asymmetric muscle weakness. All of them were NLSDM caused by *PNPLA2* mutation, suggesting that the clinical manifestations were asymmetrical muscle weakness and the muscle pathology was intramuscular fat storage. These two patients need to consider the possibility of NLSDM. The symptoms of muscle weakness in patients with LSM are often fluctuating, aggravated by the inability to resolve symptoms after exercise. In half of the patients, muscle weakness symptoms are aggravated under stress conditions such as infection, fatigue, cold, and hunger; thus, the lifestyle intervention for LSM patients is equally important. Long-term use of low-dose riboflavin can avoid the recurrence of the symptoms, such as muscle weakness. The LSM patients had mild to moderate elevation of muscle enzymes and an increasing trend of CK. Most patients in this group had elevated serum CK levels, with soft to medium height, suggesting damage to muscle cells.

In addition to muscle involvement performance, LSM patients may be linked to multiple system involvement [19, 20]. In this article, five subjects had sinus tachycardia, and rare T-wave changes were observed. A few patients start with gastrointestinal symptoms such as nausea and vomiting, which may be related to lipid storage in gastrointestinal mucosal, or a metabolic disorder may have occurred [20, 21]. Most patients with elevated uric acid may be associated with the following causes: 1. Muscle exercise caused elevated purine metabolite concentrations in the plasma by increasing the flow of purine nucleotides from muscle tissue to plasma, leading to hyperuricemia due to increased uric acid synthesis (myogenic hyperuricemia) [22]; 2. Other organic acids in the body, such as lactic acid, pyruvic acid, etc., inhibit the secretion of uric acid by the renal tubules, resulting in reduced uric acid excretion [23]. Blood and urine organic acid tests in six patients with LSM showed that three patients developed urinary symptoms of increased type 2 plus type 3 hydroxyglutaric acid. Besides, RR-MADD is often combined with an increase in blood organic acid content, indicating the existence of organic acids that interfere with the possibility of uric acid excretion. However, patients with non-RR-MADD also have elevated uric acid, suggesting that other pathways lead to elevated uric acid that requires further investigation. Electromyography provides an essential basis for the diagnosis of muscle disease. LSM electromyography is mainly characterized by myogenic damage, but individual patients may also have neurogenic damage or mixed. The electromyography of this group of patients met the above characteristics.

Muscle biopsy is an essential means of diagnosing LSM. LSM muscle pathology is characterized by abnormal storage of lipid droplets in muscle fibers, mainly involving type I fibers [24]. Many small circular vacuoles can be seen in the HE-stained muscle fibers. In severe cases, the fused large vacuoles can be seen, and the muscle fibers have a broken appearance. ORO staining shows that the vacuoles in the muscle fibers are fat deposits, and both types of muscle fibers can be involved, with type I muscle fibers. However, LSM exact typing needs to rely on genetic testing [25]. The *ETFDH* gene consists of 13 exons and encodes 617 amino acids, including the FAD and ubiquinone domains. Most of the mutations are found in the FAD domain [26, 27]. The most common mutations in the *ETFDH* gene in this group were c.770A > G and c.1227A > C, and at least one mutation site was located in the FAD domain, which was consistent with the diagnosis of RR-MADD. After repeated treatment with riboflavin and coenzyme Q10 in patients with RR-MADD, the clinical symptoms were significantly improved. This study is similar to Juan Wang et al. use of the same method in a case report to treat patients with RR-MADD with ETFDH mutations to achieve equivalent therapeutic effects. However, the measurement and method of using coenzyme Q10 are different from this study [28]. Some patients reviewed muscle pathology and found that the muscle fibers’ lipid droplets decreased or even disappeared. LSM caused by primary carnitine deficiency is a lipodystrophy myopathy with fatigue and muscle weakness as the primary clinical manifestations. The pathological changes mainly accumulate lipid droplets in muscle fibers, which generally do not cause apparent mitochondrial structure abnormal. In this study, medical staff should promptly supplement carnitine with muscle weakness during patients’ treatment. Later, the patient’s weakness gradually improved. However, the clinical symptoms of LSM patients treated with oral carnitine can be moderately improved or ultimately return to normal. However, this treatment method does not supplement muscle carnitine storage, and we recommend it as an adjuvant treatment for patients with LSM [25, 29].

The disease of LSM is an autosomal recessive inheritance. From a genetic point of view, a homozygous mutation or a compound heterozygous mutation can cause disease. A patient with a single heterozygous mutation is a gene carrier and does not cause disease. In this article, five patients were single heterozygous mutations, combined with their clinical manifestations and pathological features consistent with LSM diagnosis. Four patients with *ETFDH* gene mutations were relieved of muscle weakness after vitamin B2, considering LSM diagnosis precise. Similar reports have been reported for patients with a single heterozygous mutation [27], considering that the alleles of the possible mutation sites are incompletely inactivated, that the mutation site is located outside the exon, or that we are unknown. Other types of mutations are for further study.

## 5. Conclusion

The clinical LSM patients presenting multiple manifestations, mainly proximal limb muscle weakness and exercise intolerance, may be associated with trunk muscle weakness and numerous systems. CK increased to varying degrees, combined with elevated uric acid. LSM muscle pathology is abnormal storage of lipid droplets in muscle fibers, mainly involving type I fibers. The LSM patients in this group are mainly MADD caused by mutation of the *ETFDH* gene. The most common mutations are c.770A > G and c.1227A > C. RR-MADD has a good prognosis, so it is necessary to perform an accurate typing diagnosis of LSM.

## Figures and Tables

**Table 1:** Summary of gene mutations related to LSM.

NO.	*Gene*	Exon	Nucleotide change	Amino acid change	Hom/Het
1	*ETFDH*	exon3	c.250G > A	p.A84T	het
	*ETFDH*	exon12	c.1531G > A	p.D511N	het
2	*ETFDH*	exon7	c.770A > G	p.Y257C	het
	*ETFDH※*	exon12	c.1534G > A	p.G512R	het
	*ETFDH※*	exon12	c.1552C > G	p.L518	het
3	*ETFDH*	exon7	c.770A > G	p.Y257C	het
	*ETFDH*	exon12	c.1531G > A	p.D511N	het
4	*ETFDH*	exon3	c.380T > G	p.L127R	het
	*ETFDH*	exon10	c.1227A > C	p.L409F	het
5	*ETFDH※*	exon3	c.176-1G > A	Splicing	het
	*ETFDH*	exon10	c.1211T > C	p.M404T	het
6	*ETFDH*	exon10	c.1227A > C	p.L409F	het
	*ETFDH*	exon11	c.1395T > G	p.Y465X	het
7	*ETFDH*	exon3	c.389A > T	p.D130V	het
	*ETFDH※*	exon10	c.1123C > A	p.P375T	het
8	*ETFDH*	exon2	?		het
	*ETFDH*	exon10	c.1227A > C	p.L409F	het
	*ETFDH*	exon10	c.1281-1282delAA		het
9	*ETFDH*	exon3	c.389A > T	p.D130V	het
	*ETFDH※*	exon10	c.1285+2T > C	Splicing	het
10	*ETFDH*	exon10	c.1211T > C	p.M404T	het
	*ETFDH※*	Exon 1–5	Lose		het
11	*ETFDH*	exon1	c.67G > A	p.A23T	het
	*ETFDH*	exon5	c.587A > G	p.Y196C	het
	*ETFDH※*	exon10	c.1351G > A	p.G451R	het
12	*PNPLA2*	exon6	c.757+1G > T	Splicing	hom
13	*ETFDH*	exon3	c.250G > A	p.A84T	het
	*ETFDH※*	exon5	c.511A > G	p.N171D	het
14	*ETFDH※*	exon4	c.470C > T	p.P157L	het
	*ETFDH*	exon7	c.770A > G	p.Y257C	het
15	*ETFDH*	exon7	c.770A > G	p.Y257C	het
	*ETFDH*	exon10	c.1227A > C	p.L409F	het
16	*ETFDH*	exon5	c.524G > A	p.R175H	het
17	*PNPLA2※*	exon7	c.863C > G	p.S288W	het
18	*ETFA※*	exon8	c.649A > G	p.T217A	het
	*ETFDH※*	exon10	c.1285+1G > A	Splicing	het
19	*ETFA※*	exon8	c.649A > G	p.T217A	het
	*ETFDH※*	exon10	c.1285+1G > A	Splicing	het
20	*ETFDH*	exon13	c.1691-3C > G	Splicing	het

Het: heterozygote; HOM: homozygote; ※: Novel mutations; ?: Unknown mutation because of information absence.

**Table 2:** The clinical findings, symptoms, and laboratory findings.

Factor	Patients (n = 20)	Mean ± SD
Demographic characteristics		
Gender (male/female)	10/10 (50%)	
Age (years)	8–48	26.1 ± 2.9
Medical history	3/20 (15.0%)	
Course of disease	1m–7y	
Family medical history	3/20 (15.0%)	
Sporadic case	17/20 (85.0%)	
Chronic course of disease (years)	1–7	
Cardinal symptom		
Lower extremity weakness	8/20 (40.0%)	
Limb weakness	7/20 (35.0%)	
Upper limb weakness	3/20 (15.0%)	
Poor appetite	1/20 (5.0%)	
Exercise intolerance	12/20 (60.0%)	
Masticatory muscle weakness	6/20 (30.0%)	
Dysphagia	2/20 (10.0%)	
Respiratory muscle weakness	2/20 (10.0%)	
Myalgia	6/20 (30.0%)	
Decreased muscle strength	16/20 (80.0%)	
Weakness when lifting the head	5/20 (25.0%)	
Symmetrical muscle weakness	15/20 (75.0%)	
Asymmetric muscle weakness	2/20 (10.0%)	
Proximal muscle weakness	11/20 (55.0%)	
Distal weakness	1/20 (5.0%)	
proximal and distal weakness	7/20 (35.0%)	
Sinus tachycardia	5/20 (25%)	
Nausea and vomiting	5/20 (25%)	
Inducing factor		
Fatigue	6/20 (30.0%)	
Infected	2/20 (10.0%)	
Catch a cold	1/20 (5.0%)	
Abrosia	1/20 (5.0%)	
Laboratory testing		
Serum creatine kinase elevated	17/19 (89.5%)	3753.71 ± 6156.26 U/L
Uric acid elevated	8/10 (40.0%)	655.30 ± 351.80 U/L
Electromyography		
Myogenic damage	10/16 (62.5%)	
Neurogenic damage	5/16 (31.3%)	
Blood and urine metabolic screening		
Glutaric aciduria	3/6 (50.0%)	
Increased lipoylcarnitine	1/6 (16.7%)	
